# Expanded progenitor populations, vitreo-retinal abnormalities, and Müller glial reactivity in the zebrafish *leprechaun/patched*2 retina

**DOI:** 10.1186/1471-213X-9-52

**Published:** 2009-10-19

**Authors:** Jonathan Bibliowicz, Jeffrey M Gross

**Affiliations:** 1 Section of Molecular Cell and Developmental Biology, The University of Texas at Austin, Austin, TX, USA; 2 Institute of Cell and Molecular Biology, The University of Texas at Austin, Austin, TX, USA; 3 Institute for Neuroscience, The University of Texas at Austin, Austin, TX, USA

## Abstract

**Background:**

The roles of the Hedgehog (Hh) pathway in controlling vertebrate retinal development have been studied extensively; however, species- and context-dependent findings have provided differing conclusions. Hh signaling has been shown to control both population size and cell cycle kinetics of proliferating retinal progenitors, and to modulate differentiation within the retina by regulating the timing of cell cycle exit. While cell cycle exit has in turn been shown to control cell fate decisions within the retina, a direct role for the Hh pathway in retinal cell fate decisions has yet to be established *in vivo*.

**Results:**

To gain further insight into Hh pathway function in the retina, we have analyzed retinal development in *leprechaun/patched2 *mutant zebrafish. While *lep/ptc2 *mutants possessed more cells in their retinas, all cell types, except for Müller glia, were present at identical ratios as those observed in wild-type siblings. *lep/ptc2 *mutants possessed a localized upregulation of GFAP, a marker for 'reactive' glia, as well as morphological abnormalities at the vitreo-retinal interface, where Müller glial endfeet terminate. In addition, analysis of the over-proliferation phenotype at the ciliary marginal zone (CMZ) revealed that the number of proliferating progenitors, but not the rate of proliferation, was increased in *lep/ptc2 *mutants.

**Conclusion:**

Our results indicate that Patched2-dependent Hh signaling does not likely play an integral role in neuronal cell fate decisions in the zebrafish retina. *ptc2 *deficiency in zebrafish results in defects at the vitreo-retinal interface and Müller glial reactivity. These phenotypes are similar to the ocular abnormalities observed in human patients suffering from Basal Cell Naevus Syndrome (BCNS), a disorder that has been linked to mutations in the human *PTCH *gene (the orthologue of the zebrafish *ptc2*), and point to the utility of the *lep/ptc2 *mutant line as a model for the study of BCNS-related ocular pathologies. Our findings regarding CMZ progenitor proliferation suggest that, in the zebrafish retina, Hh pathway activity may not affect cell cycle kinetics; rather, it likely regulates the size of the retinal progenitor pool in the CMZ.

## Background

During retinal development, proliferation and differentiation must be tightly coordinated in order to produce a tissue of the proper size and containing the correct cell types [1]. The Hh pathway has been shown to play a critical role in controlling these two seemingly opposite processes [2]. Early in retinal development, the optic vesicle is composed of a population of proliferating neuroepithelial cells that will ultimately give rise to the mature retina [3]. Differentiation of the retinal neuroepithelium occurs in a succession of temporally overlapping waves [4]. In the zebrafish, the first cells to exit the cell cycle become retinal ganglion cells (RGCs), which differentiate in a Sonic Hh (Shh)-dependent wave [5]. A second Hh-dependent wave of differentiation in the inner nuclear layer (INL) occurs almost simultaneously with the first wave, and is responsible for the differentiation of the multiple cell types of the INL (horizontal, amacrine, and bipolar cells, and Müller glia) and the rod and cone photoreceptors of the outer nuclear layer (ONL) [6]. In addition, extra-retinal Hh signaling originating in the retinal pigment epithelium (RPE) has been suggested to direct photoreceptor differentiation [7]. While the role of the Hh pathway in cell cycle exit and differentiation of retinal progenitors is well described, comparatively less is known about its possible influence on cell fate decisions. In *Xenopus*, over-expression of p27Xic1, which promotes cell cycle exit, results in increased numbers of early-born cell types (RGCs), while the over-expression of cyclin E1, which inhibits cell cycle exit, biases cell fate towards late-born cell types (e.g. Müller glia) [8]. Similarly, Shh has been shown to promote early cell cycle exit in the *Xenopus *retina [9]; however, a direct role of the Hh pathway in dictating retinal cell fate has yet to be established *in vivo*.

While the cells of the central retina of the zebrafish exit the cell cycle by 60 hours post fertilization (hpf) [10], a population of retinal progenitors at the CMZ is maintained and continues to proliferate throughout the animal's lifetime [11,12]. The spatial pattern of cells within the CMZ, with retinal stem cells at the most peripheral region followed by proliferative retinal progenitors and finally differentiating progenitors more centrally, is thought to recapitulate the temporal sequence of early retinal development [13]. Some of the factors that control early retinal development, such as *notch, rx1, pax6a, and vsx2/chx10*, are expressed in the zebrafish CMZ [11]. In *Xenopus*, expression of *gli2*, *gli3*, and *smoothened*, is found at the retinal margin, suggesting a role for the Hh pathway in the stem cell/progenitor population in the CMZ [14]. Shh over-expression studies in chick support a role for the Hh pathway as a mitogen in the CMZ [15], consistent with its described mitogenic effects on retinal progenitors in early zebrafish and mouse retinal development [16,17].

Invaluable knowledge regarding Hh function in the developing retina has been gained from the analysis of Hh pathway mutants in zebrafish. For example, the zebrafish mutants *syu *(*shh*) and *smu *(*smoothened*) have helped elucidate the complex mechanisms of Hh-dependent neural differentiation and proliferation [7,16]. However, retinal differentiation is severely defective or altogether absent in these mutants due to defective Hh signaling, making it difficult to arrive at definitive conclusions about the possible role of the Hh pathway in cell fate decisions. To address this issue, we analyzed retinal development in the zebrafish *lep/ptc2 *mutant, in which the Hh pathway is 'over-active'. *lep/ptc2 *mutants possess a non-sense mutation in the exon encoding the sixth trans-membrane domain of Patched2 [18], which is predicted to abolish its function [18,19]. Loss of Patched function results in an 'over-active' Hh pathway due de-repression of Smoothened [20]. Normally, in the absence of Hh ligand, the Patched protein inhibits the activity of Smoothened. Binding of the Hh ligand to its Patched receptor relieves this inhibition, ultimately resulting in increased transcription of Hh target genes [21,22]. A non-functional Patched would therefore result in loss of inhibition on, and constitutive activity of, Smoothened in *ptc2 *deficient cells. *lep/ptc2 *mutants possess increased proliferation in multiple tissues including the retinal CMZ [18]. Utilizing this mutant as a tool, we sought to gain further insight into Hh function with respect to retinal cell fate decisions and to determine how Hh activity influences progenitor proliferation at the CMZ. Our results revealed no significant change in neuronal composition within the retina, but we did note a reduction in differentiated Müller glia that was accompanied by localized Müller glial reactivity and abnormalities at the vitreo-retinal interface. In addition, the results of cell cycle analyses suggest that the over-proliferation at the CMZ likely results from an expansion of the progenitor cell population, and not from direct effects of the mutation on cell cycle kinetics within progenitor cells.

## Results

### lep/ptc2 mutants possess enlarged retinas and overgrown irises, while lens and RPE morphology are largely normal

**Figure 1:**
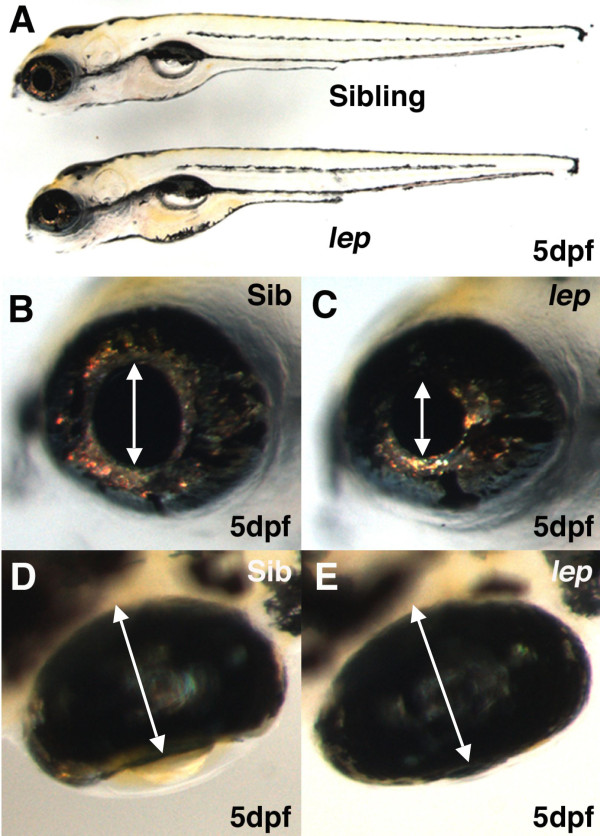
***lep/ptc2 *mutants possess enlarged retinas and reduced pupil size**. Whole embryo lateral view shows that *lep/ptc2 *embryos are largely normal compared to their siblings (A). High magnification images of sibling (B) and *lep/ptc2 *(C) eyes reveal reduced pupil size in *lep/ptc2 *(arrows in B, C). Dorsal view images illustrate the overgrowth of retinal tissue as the retina is thicker and appears to engulf the lens (arrows in D, E).

**Figure 2:**
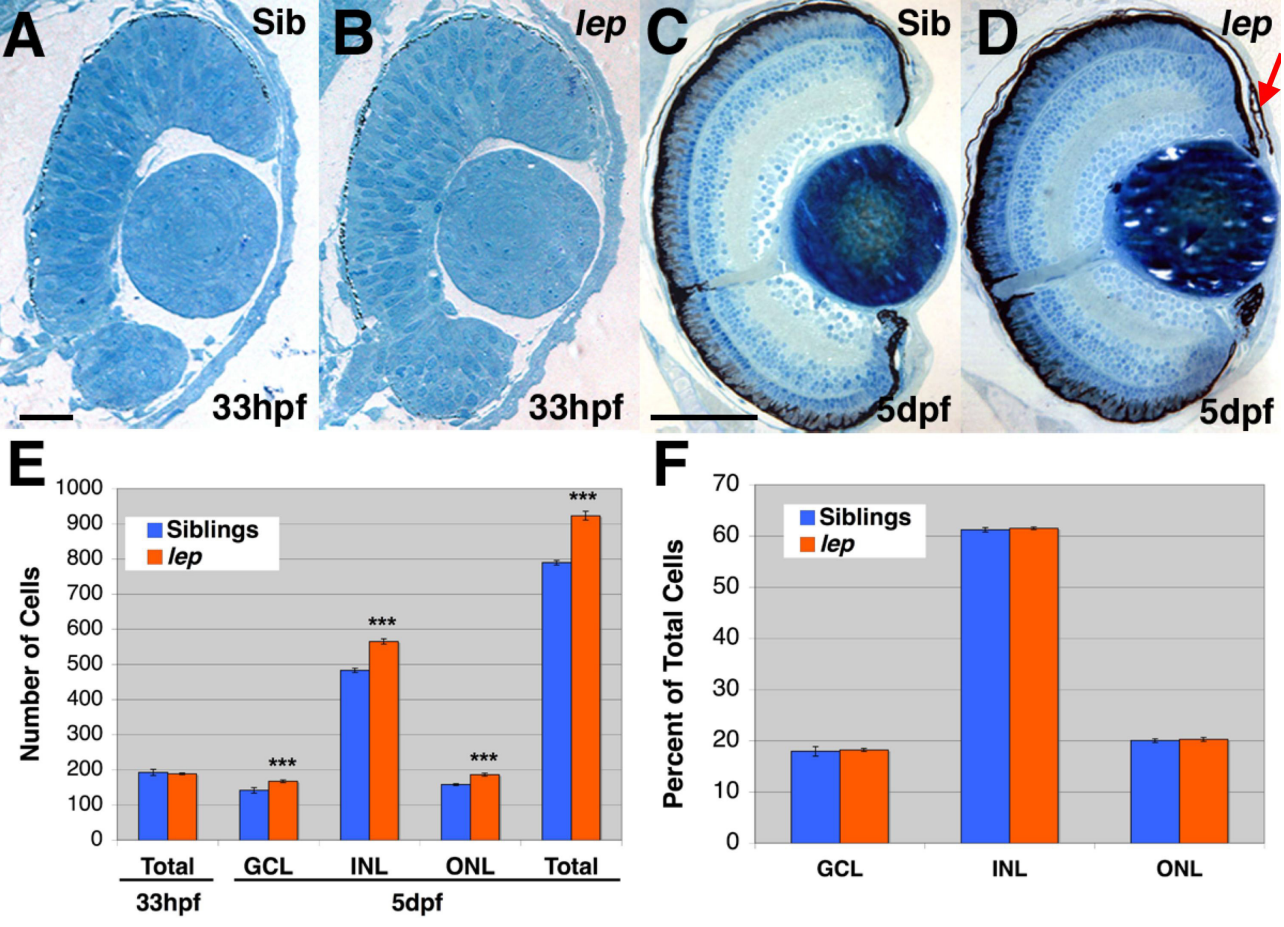
***lep/ptc2 *mutant retinas possess proportional increases in cell numbers in all three nuclear layers**. Histology of sibling (A, C) and *lep/ptc2 *mutant (B, D) retinas at 33hpf (A, B) and 5dpf (C, D). E) Quantification of total number of retinal cells at 33hpf and 5dpf, and of cells in each nuclear layer at 5dpf (18.0%, 17.0%, and 17.8% increases in GCL, INL, and ONL, respectively. ***p < 0.0001). F) Graph of the proportion of each nuclear layer as a percentage of total retinal cells at 5dpf. Scale Bars = 20 um in A, B and 100 um in C, D.

*lep/ptc2 *mutants were isolated based on expanded PCNA staining in multiple tissues including the retinal CMZ [18]. While mutant embryos appear to be of normal size at 5 days post fertilization (dpf) (Figure 1A), their retinas are enlarged (Figure 1D, E) and they possess reduced pupil size ([18] and Figure 1B, C). To characterize ocular tissues in *lep/ptc2 *mutants, we performed histological analyses at 33 hpf, around the time of the onset of retinal differentiation, and at 5dpf, when the laminar organization of the embryonic retina is complete [10,23]. *lep/ptc2 *retinas appear largely normal at 33dpf and do not possess increases in retinal cell number relative to phenotypically wild-type siblings (Figure 2A, B, E). At 5dpf, however, *lep/ptc2 *retinal cells are more tightly packed and mutant retinas contain a larger number of cells as compared to their phenotypically wild-type siblings (Figure 2C, D, E). Normal retinal lamination of differentiated neurons has been shown to be dependent on Shh signaling *in vitro *[24]. Lamination is largely normal in *lep/ptc2 *mutants and all major cell types are present (Figure 2C, D). Loss-of-function experiments in both frog and mouse have suggested a role for the Hh pathway in the development of the RPE [14,25]. In *lep/ptc2 *mutants the RPE is of normal thickness and morphology. The Hh pathway has also been implicated in lens development [26,27], and the reduced pupil size present in *lep/ptc2 *mutants was previously attributed to potential degeneration of the lens [28]. No gross morphological defects are evident in the lenses of *lep/ptc2 *mutants at either 33hpf or 5dpf, however. Additionally, histological analyses revealed an abnormality in the anterior segment of *lep/ptc2 *eyes where the iris was over-grown and extended further over the lens than in sibling retinas (Figure 2D). The enlarged iris and the overgrowth of the CMZ are the likely causes of the decreased pupil size, rather than defects in lens morphology.

### Neuronal composition of the lep/ptc2 retina is normal, while Müller glia are under-represented

Hh signaling has been shown to be required for the differentiation of multiple cell types in the vertebrate retina [6,7,29] and is thought to promote cell fate decisions by modulating the timing of cell cycle exit [30]. Shh gain-of-function experiments in *Xenopus *link a faster cell cycle with early cell cycle exit [9], which in turn results in an over-production of early-born retinal cell types at the expense of late-born cell types [8]. We therefore reasoned that in *lep/ptc2 *retinas, where the Hh pathway is over-active, early-born cell types would be over-represented while late-born cell types would be reduced in number. Cell counts from histological sections of *lep/ptc2 *mutants revealed a statistically significant increase in cell number in all three retinal nuclear layers (GCL, INL, and ONL), as well as in total retinal cells, when compared to those from phenotypically wild-type siblings (Figure 2E). However, no significant change in the proportion of each layer was observed when the number of cells per layer was calculated as a percentage of total retinal cells (Figure 2F). This suggests that all three retinal cell layers are proportionally increased in *lep/ptc2 *retinas.

**Figure 3:**
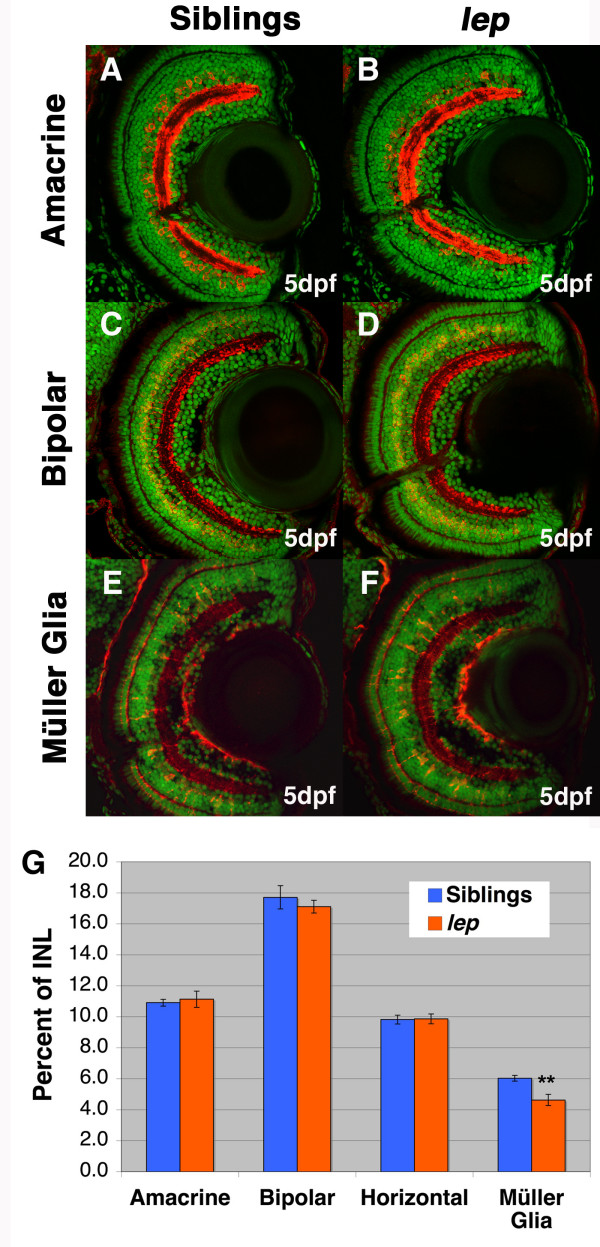
**All neuronal cell types in the *lep/ptc2 *INL are increased proportionately, while Müller glia are reduced**. Immunohistochemistry of sibling (A, C, E) and *lep/ptc2 *(B, D, F) retinas for Amacrine cells (A, B), Bipolar cells (C, D), and Müller glia (E, F). G) Cell counts for each INL cell type. No significant change is cell number in all neuronal cell types when calculated as a percentage of total INL cells. Müller glia, however, were reduced in *lep/ptc2 *retinas by 29% relative to sibling retinas (**p = 0.0013).

**Figure 4:**
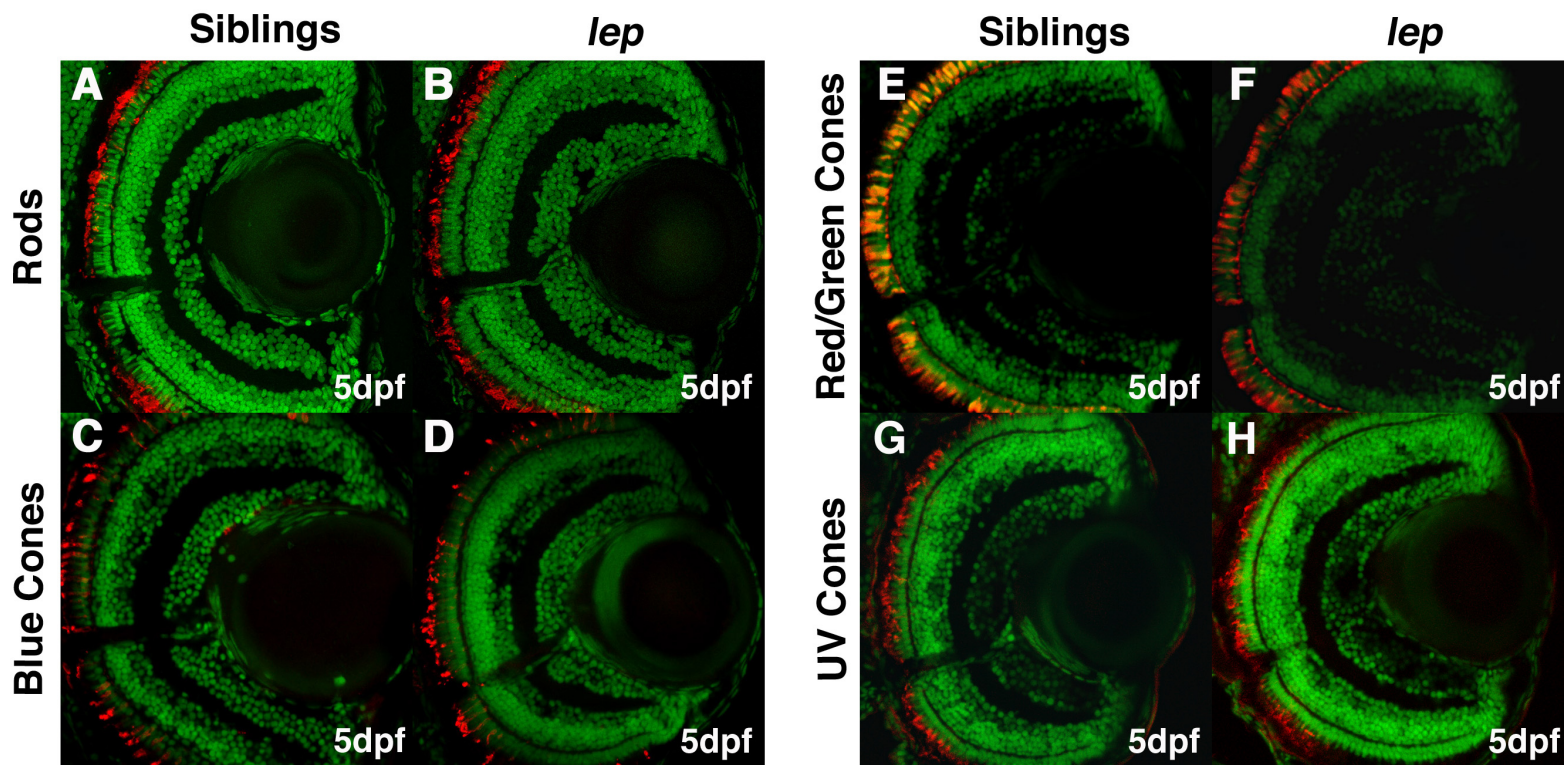
**Photoreceptors appear unaffected in *lep/ptc2 *retinas**. Immunohistochemical analysis of rods (A, B), blue cones (C, D), red/green cones (E, F), and UV cones (G, I) in sibling (A, C, E, G) and *lep/ptc2 *retinas (B, D, F, I). All photoreceptor types were present and appear normal.

It is possible that in *lep/ptc2 *mutants, an overactive Hh pathway could alter neuronal composition within each layer. To test this hypothesis, immunohistochemistry using markers for each specific cell type in the INL and ONL was performed on retinal sections at 5dpf, when all cell types are normally differentiated and the embryonic retina is fully developed. Since the GCL layer is almost exclusively composed of retinal ganglion cells (RGCs), no further analysis was performed on this layer. In order to analyze cell types within the INL, immunohistochemistry was performed using antibodies that mark amacrine cells (5E11), bipolar cells (PKC) and Müller glia (Glutamine Synthetase, GS), while horizontal cells were detected according to their stereotypical morphology in histological sections. This analysis revealed that while the proportion of each neuronal cell type in the INL was not significantly changed in *lep/ptc2 *mutants, there was a statistically significant decrease in the proportion of differentiated Müller glia (Figure 3). For the ONL, immunohistochemistry was performed to detect rods (Zpr3) and each three cone opsins (blue, red/green, and UV) [31]. Due to the diffuse nature of the staining of some of the markers used, precise cell counts were not performed on these cell types. However, immunohistochemisty did show that these cell types are present in *lep/ptc2 *and qualitatively appear unaffected (Figure 4). In all, *lep/ptc2 *mutants possess more cells in their retinas and each cell type, except for Müller glia, was present at identical ratios to those observed in phenotypically wild-type siblings.

### lep/ptc2 mutants possess abnormalities at the vitreo-retinal boundary and localized Müller glial reactivity

**Figure 5:**
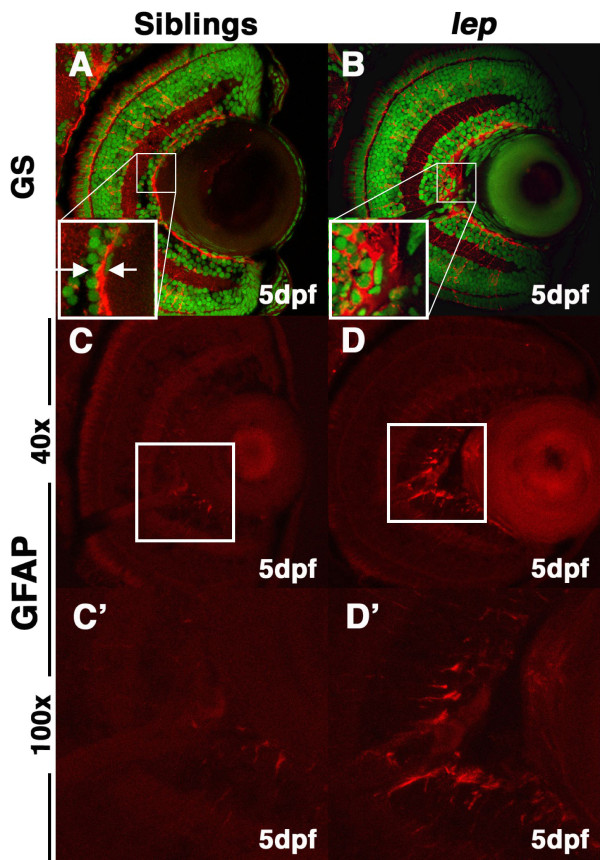
***lep/ptc2 *mutants display Müller glial reactivity and morphological abnormalities in the ILM**. Immunohistochemical analysis using glutamine synthetase (GS) antibody, which marks differentiated Müller glia and their endfeet at the ILM, highlights disruptions in the ILM. (A) In sibling retinas, the ILM is tight and continuous (inset). (B) In *lep/ptc2 *retinas the ILM is discontinuous and Müller glial endfeet do not terminate properly at the ILM (inset). Glial fibrillary acidic protein (GFAP) antibody staining reveals elevated immuno-reactivity in the inner retina, adjacent to the optic nerve of *lep/ptc2 *(D and arrows in D') mutant retinas as compared to siblings (C, C'). Approximately 40% of analyzed mutants displayed significant ILM disruptions and elevated GFAP levels.

Müller glial endfeet terminate at, and contribute to, the inner limiting membrane (ILM), which separates the retina from the vitreous [32]. Interestingly, our immunohistochemical analysis of differentiated Müller glia revealed disruptions in the integrity of the ILM (Figure 5A, B). Since humans who possess mutations in the *PTCH *gene (orthologue of the zebrafish *ptc2 *[33]) have been previously shown to possess abnormalities in the vitreo-retinal interface [34], we sought to further investigate this phenotype. High magnification examination of GS-stained retinas revealed disruptions in the integrity of the ILM in 40% of the *lep/ptc2 *retinas examined. While Müller glial endfeet form a tight and continuous boundary at the vitreo-retinal boundary in sibling retinas, the ILM in *lep/ptc2 *retinas was often discontinuous and disorganized, and endfeet did not appear to properly terminate in the ILM (Figure 5A, B). In *Ptc1*^+/- ^mice, ocular abnormalities are accompanied by the upregulation of GFAP, a marker of reactive Müller glia, and ectopic proliferation of Müller glia in the central retina [34].

Immunohistochemical analysis of GFAP revealed that its levels in Müller glia were indeed higher in *lep/ptc2 *mutant retinas as compared to their phenotypically wild-type siblings (Figure 5C, C', D, D'). Higher levels of GFAP were often localized to Müller glial endfeet in the central retina, adjacent to the optic nerve. However, BrdU incorporation assays in *lep/ptc2 *mutants at 5dpf did not reveal ectopic proliferation in the central retina (see Additional file 1), suggesting that the upregulation of GFAP is not coupled with Müller glial proliferation in this context. In addition, the localized nature of Müller glial reactivity in not due to abnormal cell death in the *lep/ptc2 *retina and/or optic nerve since no apoptotic nuclei were detected by TUNEL assays (see Additional file 1).

### patched2 (ptc2) is expressed in the progenitor/stem cell populations of the CMZ

**Figure 6:**
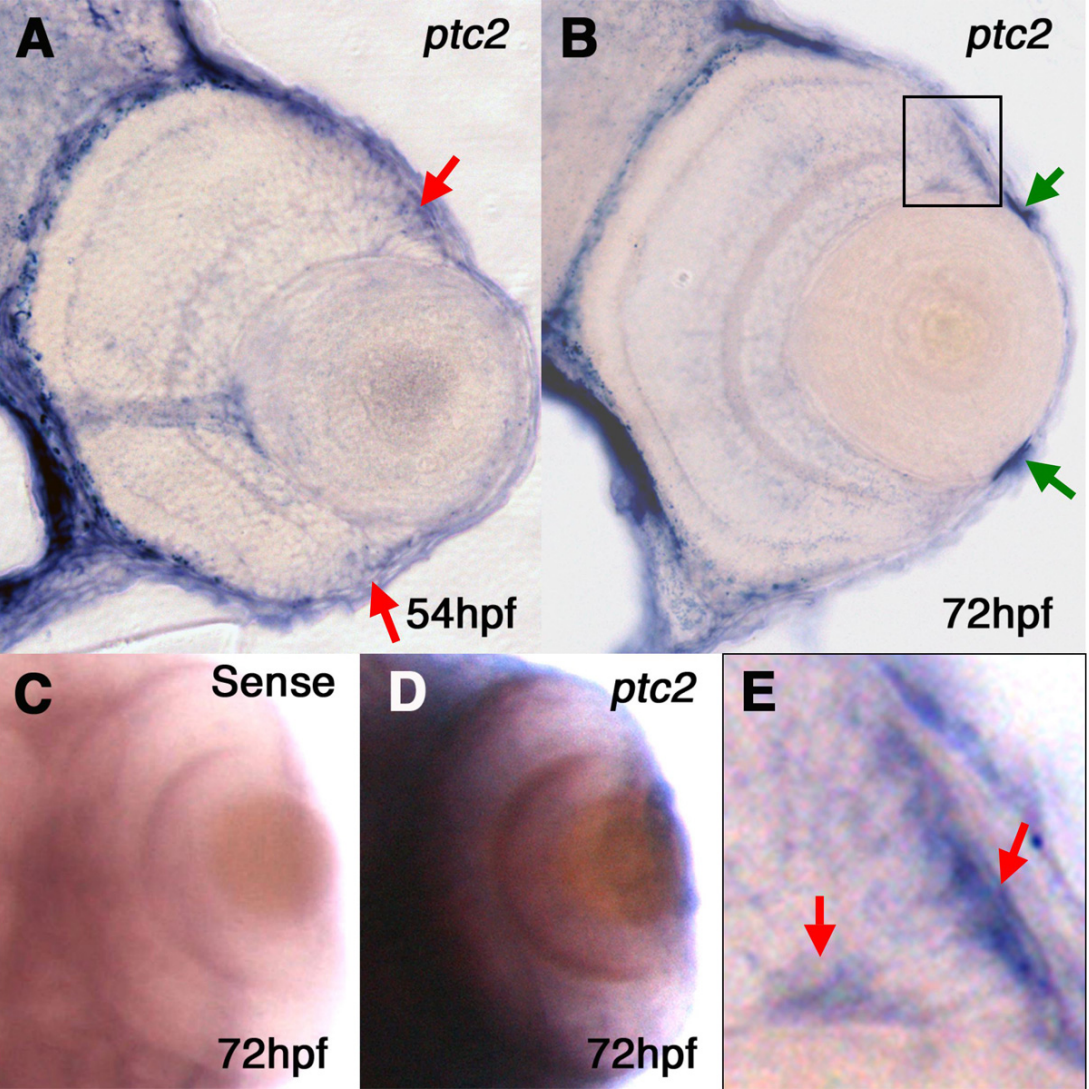
***ptc2 *is expressed at the zebrafish CMZ**. *In-situ *hybridization for the *ptc2 *transcript shows expression at the retinal margin at 56hpf (arrows in A). At 72hpf, transcript is detected throughout the CMZ (B) and at the iridio-corneal angle (green arrows), with high magnification of CMZ region (E) revealing more robust staining adjacent to the RPE and in a region just apposed to the GCL (red arrows). Whole mount images from a dorsal view of sense (C) and anti-sense (D) probes *in-situ *hybridization show that staining is specific.

We next sought to better understand the nature of the over-proliferation at the CMZ by defining the retinal cell populations that express the *ptc2 *transcript. At 56hpf, prior to the time that the overgrowth phenotype becomes detectable, *ptc2 *expression was visible at the retinal margin, corresponding to the undifferentiated population of retinal progenitors of the CMZ (Figure 6A). Expression continued to be restricted to the CMZ at 72hpf (Figure 6B), consistent with previously reported expression of Hh target genes in the *Xenopus *CMZ [14]. Closer inspection of *ptc2 *expression in the CMZ at 72hpf revealed that low-level *ptc2 *expression was detected throughout most of the CMZ, consistent with previous reports [35]. Staining was more robust in two distinct CMZ cell populations; the first was immediately apposed to the RPE and the second was a patch of cells at the edge of the CMZ, bordering the differentiated GCL (arrows in Figure 6E). Hh ligands have been shown to be expressed in both the RPE [14] and the GCL [5,24] during retinal development, and Patched genes are expressed in a Hh-dependent manner [33]. Thus, these CMZ regions may reflect areas of active Hh signaling. Finally, the *ptc2 *transcript is expressed at the iridio-corneal angle, where the iris and cornea converge [36] (arrows in Figure 6B).

### The number of proliferating progenitors, but not the rate of progenitor proliferation, is increased in the lep/ptc2 CMZ

**Figure 7:**
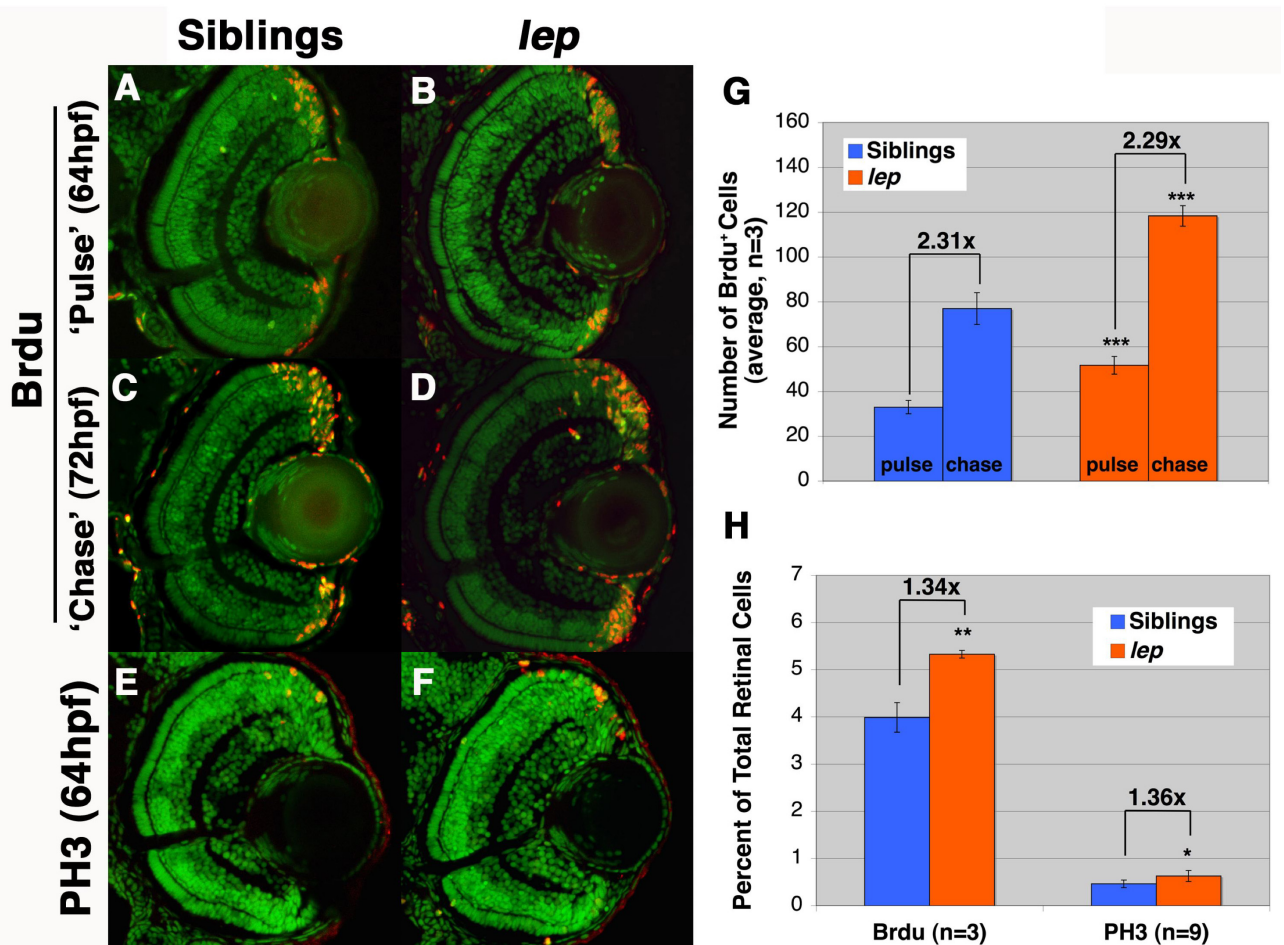
**The number of proliferating CMZ progenitors is increased in *lep/ptc2*, while proliferation rate is unaffected**. (A, B) BrdU 'pulse' in sibling (A) and *lep/ptc2 *mutants (B). BrdU^+ ^cells are observed throughout the CMZ. (C, D) After an 8 hour 'chase', BrdU^+ ^cells are observed at the CMZ, as well as within the retina of sibling (C) and *lep/ptc2 *mutants (D). E, F) PH3 labeling in sibling (E) and *lep/ptc2 *(F) at 64hpf. G) Quantification of BrdU^+ ^cells in sibling and *lep/ptc2 *after 'pulse-chase' experiment. *lep/ptc2 *retinas contained 67% more BrdU^+ ^cells than sibling retinas at 64hpf. After an 8 hour 'chase', the number of BrdU^+ ^cells increased by almost identical ratios in siblings (2.31-fold increase) and *lep/ptc2 *mutants (2.29-fold increase, ***p < 0.0001). H) Quantification of BrdU- and PH3-positive cells as a percentage of total retinal cells in lep/ptc2 and wild-type siblings at 64hpf. The proportion of both BrdU^+ ^and PH3^+ ^cells increased by similar ratios (1.34-fold increase, **p < 0.001 and 1.36-fold increase, *p < 0.01, respectively).

*Ptc1*^+/- ^mice possess increased numbers of progenitor cells at all stages of retinal development, suggesting a role for the Hh pathway in controlling the size of retinal progenitor populations in the ciliary margin, and these mice also possess a persistent progenitor population in the retinal margin as adults [37]. Studies in post-hatch chicks suggest that Shh regulates the proliferation of CMZ progenitors [15]. In addition, Shh over-expression studies in *Xenopus *have revealed a role for the Hh pathway in regulating the length of the cell cycle in the progenitor populations of the CMZ [9]. Given the differing roles for Hh pathway activity in these contexts, we wanted to determine whether the over-proliferation in *lep/ptc2 *retinas was due to an increase in the number of proliferating cells and/or an increase in the proliferation rate of the progenitor cell population in the CMZ. To address these possibilities, we first performed a 'pulse-chase' BrdU analysis on *lep/ptc2 *and sibling embryos starting at 64hpf, when a mild retinal phenotype is first detectable. Embryos were sorted according to phenotype and exposed to a short pulse (15 min) of BrdU. Half of the embryos were immediately fixed for sectioning, while the other half were transferred to non-BrdU containing fish water and fixed 8 hours later (72hpf). This enabled us to determine the rate of progenitor proliferation in both *lep/ptc2 *and sibling retinas during the 8 hour chase period. The results of these assays revealed that, on average, *lep/ptc2 *retinal sections contained 67% more BrdU-positive cells in the CMZs when compared to phenotypically wild-type sibling retinas at 64hpf (Figure 7A, B, E). At 72hpf, after the 8 hour 'chase', the average number of BrdU-positive cells in *lep/ptc2 *and phenotypically wild-type sibling retinal sections increased by almost identical ratios (2.29-fold changes in *lep/ptc2 *vs. 2.31 fold change in sibling sections, Figure 7C, D, E). From these data, we can therefore infer that, on average, each proliferating cell gave rise to 2.3 daughter cells during the 8 hour 'chase' period. To further analyze proliferation, we assayed the expression of phosphohistone H3 (PH3) and these analyses revealed that the proportion of CMZ progenitors occupying the M phase of the cell cycle increased by approximately 34% in *lep/ptc2 *retinas at 64hpf, when compared to their phenotypically wild-type siblings (Figure 7E, F, H). The degree of this increase was almost identical to that observed in progenitor cells occupying the S phase using BrdU as a marker (Figure 7A, B, H), suggesting that the length of the S and M phases was not changed relative to the other. These results support a model in which the expanded CMZ of *lep/ptc2 *mutants arises from an expansion of the progenitor cell population therein, and not from changes in the length of the cell cycle in these progenitor cells.

## Discussion

### Retinal patterning is normal in lep/ptc2 mutants

Characterization of *lep/ptc2 *retinas with respect to cell type composition revealed that over-activity of the Hh pathway did not affect retinal patterning. Cell count analysis of all major retinal neuronal cell types revealed no change in the proportion of each cell type. This finding was surprising in light of the well established role of the Hh pathway in patterning and cell fate decisions throughout the CNS in a number of model systems [20,38,39], as well as studies in *Xenopus *which show that the over-expression of Shh results in early cell cycle exit [9], and that can, in turn, influence cell fate decisions [8]. While mouse *Ptc1*^+/- ^(the orthologue of the zebrafish *ptc2 *[33]) mutants were found to possess no defects in retinal cell fate specification, *Ptc1*^-/- ^mice could not be examined since they die *in utero *[37]. Our results indicate that Ptc2-dependent Hh signaling does not likely play an integral role in neuronal cell fate decisions in the zebrafish retina. These findings raise the possibility that the second zebrafish Patched protein (Patched1) might compensate for the lack of a functional Patched2. While *patched1 (blowout) *mutant retinas contain all major retinal cell types, the lack of a retinal phenotype might be due to the hypomorphic nature of the mutation [40,41]. However, this seems unlikely since *patched1 *transcripts are not expressed at detectable levels in retinal tissue throughout development (*data not shown *and [35]). The reduced number of differentiated Müller glia, a late born cell type [10], could feasibly be due to a bias towards early born cell types in *lep/ptc2 *mutants; however, this potential bias does not affect neuronal composition of the retina at 5dpf. It is possible that a transient bias towards early neuronal cell types could occur early in retinal development, but any differences could masked by neurons added from the CMZ later in development. Conversely, any bias could also be present in neuronal populations that arise at the CMZ, resulting in an unnoticeable change in neuronal composition of the 5dpf *lep/ptc2 *retina. With this in mind, it will be interesting to examine the proportion of retinal cell types in juvenile *lep/ptc2 *mutants, as some homozygous fish reach two months of age [18]. Finally, the Hh pathway could play a role in neuronal cell fate decisions in the retina through Hh ligand interactions with other Hh receptors. The Ihog Hh receptors have recently been suggested to compete with Patched for Hh binding [42] and act at levels at, or upstream of, Patched [43]. Indeed, the vertebrate Ihog homologue Cdo is expressed in the developing mouse retina [44]. In the future, it would be interesting to test whether certain aspects of retinal development, such as cell fate decisions, might be Ihog-dependent.

### lep/ptc2 mutants as a possible model for the study of BCNS-related ocular pathologies

In BCNS patients, abnormalities at the interface between the retina and the vitreous, known as epiretinal membranes (ERMs), are associated with disruptions of the ILM and are thought to result from the overproliferation and ectopic presence of multiple cell types, including glia [45]. BCNS ocular pathologies have been linked to mutations in the human *PTCH *gene [34,46,47], and have been shown to be associated with reactive Müller glia [34]. Müller glial reactivity is often marked by upregulation of GFAP, and *lep/ptc2 *mutants possessed regions of elevated GFAP levels. Elevated GFAP levels were consistently localized in the inner retina, where Müller glial endfeet terminate and contribute to the ILM. Staining with GS, which labels the Müller glial endfeet, revealed disruptions in ILM integrity in ~40% of mutant retinas assayed. In these cases, Müller glial endfeet did not terminate properly in the ILM, and the ILM was discontinuous. In some human patients, ERMs are found adjacent to retinal arteries [34]; interestingly, we consistently detected the presence of reactive glial endfeet in localized retinal regions adjacent to the optic nerve and embryonic retinal vasculature. Importantly, no increases in cell death were detected in the retina or optic nerve of *lep/ptc2 *mutants, or in Müller glia themselves (see Additional file 1), ruling out reactivity due to apoptosis. Finally, in other contexts, Müller glia reactivity is often coupled with ectopic proliferation of these cells during 'reactive gliosis' [48]. BrdU analysis did not detect ectopic proliferation in the central retinas of *lep/ptc2 *mutants (Figure 7 and Additional file 1), indicating that while a subset of these cells was reactive, they did not undergo a proliferative response. While reactive Müller glia have been shown to be associated with ERMs, it is unclear whether reactive Müller glia are the cause of these pathologies or, conversely, whether Müller glia become reactive in response to ERM formation. Further studies will be required to answer this question and to shed light on the cellular and molecular causes of ERM formation.

### The retinal progenitor population of the CMZ is expanded in lep/ptc2 mutants

Exact roles for Hh pathway activity in proliferating retinal progenitor cells remain unclear. Over-expression studies in *Xenopus *suggest a direct role for the Hh pathway in regulating proliferation by influencing the length of the cell cycle in CMZ progenitors [9]. In *Ptc1*^+/- ^mice, more retinal progenitors are allocated to the CMZ throughout development and into adulthood [37]. BrdU pulse-chase analysis of the progenitor population at the *lep/ptc2 *CMZ suggests that Patched-dependent Hh signaling controls the number of retinal progenitors in the zebrafish CMZ, and does not directly affect the length of the cell cycle in these cells. Importantly, cell counts at an earlier time-point, before the formation of the CMZ, revealed no increase in cell number in *lep/ptc2 *retinas as compared to their wild-type siblings (Figure 2E), suggesting that the increase in proliferating progenitors at the CMZ is not simply due to an earlier proliferative event. Shh has been shown to control stem cell maintenance in multiple organs, including the adult brain (reviewed in [49]). Indeed, the Hh pathway genes *smo*, *gli2*, and *gli3 *are expressed in the putative stem cell region of the *Xenopus *CMZ [14]. In light of our findings, it is possible that the increase in retinal progenitors in the *lep/ptc2 *CMZ is an indirect result of mis-regulation of the stem cell population rather than a direct effect on progenitor proliferation.

## Conclusion

The possible role for the Hh pathway in retinal cell fate decisions has yet to be established *in vivo*. Our results indicate that Patched2-dependent Hh signaling is not likely to play an integral role in dictating neuronal cell fate decisions in the zebrafish retina. In addition, ocular phenotypes in *lep/ptc2 *mutants that are similar to those found in human BCNS patients point to the utility of the *lep/ptc2 *mutant line as a model for the study of BCNS ocular pathologies. With regards to progenitor proliferation, our data support a role for Patched2-dependent Hh signaling in the control of the size of progenitor populations at the retinal CMZ, and not in regulating their rate of proliferation, during zebrafish eye development.

## Methods

### Zebrafish maintenance and strains

Zebrafish (*Danio rerio*) were maintained at 28.5°C on a 14 h light/10 h dark cycle. Embryos were obtained from the natural spawning of heterozygous carriers setup in pairwise crosses. Embryos were collected and raised at 28.5°C [50] and were staged according to [51]. *ptc2*^*tj222 *^outcrosses were obtained from the Zebrafish International Resource Center and were propagated by repeated outcrosses to AB fish. All animals were treated in accordance with provisions established at the University of Texas at Austin governing animal use and care.

### Histology

Histology was performed as described in [52]. Briefly, mutant and wild-type sibling embryos were collected and fixed overnight at 4°C in a solution of 1% (w/v) paraformaldehyde (PFA), 2.5% glytaraldehyde and 3% sucrose in phosphate in a 2% OsO_4 _solution, washed 3 × 5 min in PBS at room temperature and further dehydrated 2 × 10 min in propylene oxide and infiltrated 1-2 h in a 50% propylene oxide/50% Epon/Araldite mixture (Polysciences, Inc.). Embryos were then incubated overnight at RT in 100% Epon/Araldite resin with caps open to allow for propylene oxide evaporation and resin infiltration, embedded and baked at 60°C for 2-3 days. Sections 1-1.25 μm were cut, mounted on glass slides and stained in a 1% methylene blue/1% borax solution. Sections were mounted in DPX (Electron Microscopy Sciences) and photographed on a Leica DMRB microscope mounted with a DFC320 digital camera. For 5dpf cell counts, the number nuclei in each retinal layer were counted in ten eye sections obtained from different embryos. Horizontal cells were identified by location and morphology. Averages were analyzed and compared using a Student's t-test.

### Immunohistochemistry

Immunohistochemistry was performed as described in [53]. Briefly, embryos were collected and fixed overnight at 4°C in a 4% PFA solution in PBS. Embryos were then washed 3 × 10 min in PBS and incubated in a 25% sucrose/PBS solution for 1 h followed by 35% sucrose/PBS for 1 h. Embryos were then mounted in Tissue Freezing Medium (Triangle Biomedical Sciences, Inc.) and immediately transferred to -80°C freezer for storage. Frozen blocks were sectioned at 12 μm, mounted on gelatin coated slides, and let dry for 2 h. Slides were then re-hydrated in PBTD [0.1% Tween, 1% DMSO in 1× PBS] and then blocked using 5%NGS/PBTD for 2 h. Slides were incubated in primary antibody diluted in block in a humid chamber over-night at 4°C. When necessary, nuclear staining was obtained by applying Sytox-Green (Molecular Probes) diluted 1:10,000 in block immediately after removal of primary antibody. Slides were washed 3 × 10 min with PBTD and then applied with secondary antibody in block for 1 h. After 3 × 10 min washes in PBTD, slides were mounted with Vectashield (Vector Laboratories, Inc.) and coverslipped. Samples were stored at 4°C for up to one week and imaged on a Zeiss LSM5 Pascal laser scanning confocal microscope. The following primary antibodies were used: bipolar cells (PKC, 1:200, Santa Cruz Biotechnology), Müller glia (GFAP-zrf1, 1:100, ZIRC and GS, 1:500, Millipore), rods (zpr3, 1:100, ZIRC), green/red cones (zpr1, 1:100, ZIRC), PH3 (1:200, Upstate). Blue opsin (1:500) and UV opsin (1:1,000) antibodies were provided by David Hyde (Univerity of Notre Dame). Amacrine cell antibody (5E11, 1:100) was the gift of Jim Fadool. For cell counts, positively stained cells and nuclei were counted in four eye sections obtained from different embryos. For total cell counts at 33hpf, Sytox-Green staining was used. Averages were analyzed and compared using a Student's t-test.

### BrdU incorporation assays

Embryos were dechorionated and incubated in fish water with 10 μM 5-Bromo-2-deoxyuridine (BrdU, Sigma) and 15% DMSO at 4°C for mentioned time periods and either immediately sacrificed or washed three times in fish water and grown at 28.5°C prior to sacrifice after [54]. Embryos were processed for immunohistochemistry as in [40]. Mouse anti-BrdU was used at a 1:50 dilution and Cy3 anti-mouse secondary at a 1:200 dilution. Nuclei were counterstained with Sytox:Green (1:10,000, Molecular Probes). For cell counts, Brdu-positive cells and nuclei were counted in four eye sections obtained from different embryos. Averages were analyzed and compared using a Student's t-test.

### In-situ hybridization

Hybridizations were performed as described in [55] using digoxigenin labeled antisense RNA probes. *ptc2 *probe synthesis construct was provided by Brian Perkins (Texas A&M University).

## Authors' contributions

JB carried out all experimental procedures. JB and JMG conceived of the study, participated in its design and coordination, and drafted the manuscript. All authors read and approved the final manuscript.

## Supplementary Material

Additional file 1 ***lep/ptc2 *mutants do not possess ectopic cell death or proliferation in the central retina and/or optic stalk**. (A, B) BrdU incorporation and (C, D) TUNEL assays in 5dpf (A, C) phenotypically wild-type siblings and (B, D) *lep/ptc2 *mutants. Click here for file
