# Supplementary figures and images for: Expanded progenitor populations, vitreo-retinal abnormalities, and Müller glial reactivity in the zebrafish leprechaun/patched2 retina
Source: BMC Dev Biol. 2009 Oct 19;9:52. doi: 10.1186/1471-213X-9-52 (PMC2770046; doi:10.1186/1471-213X-9-52)

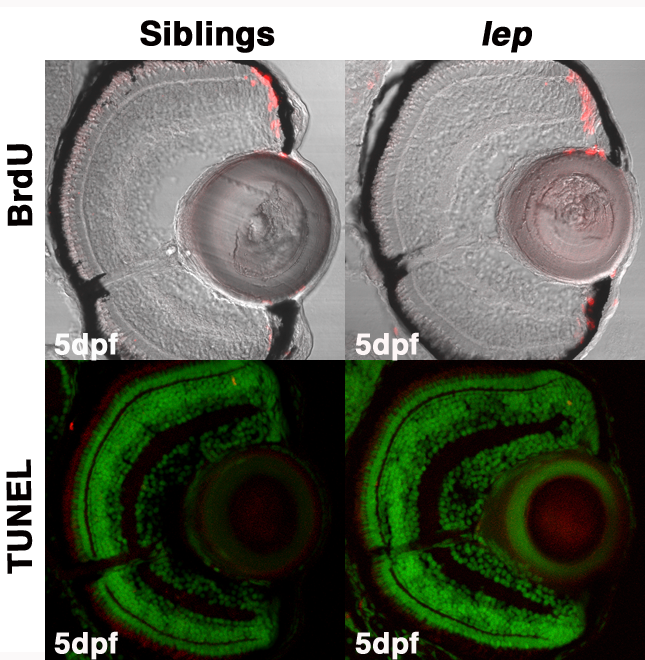

Supplement: Additional file 1 — lep/ptc2 mutants do not possess ectopic cell death or proliferation in the central retina and/or optic stalk. (A, B) BrdU incorporation and (C, D) TUNEL assays in 5dpf (A, C) phenotypically wild-type siblings and (B, D) lep/ptc2 mutants. [file 1471-213X-9-52-S1.TIFF]
